# Ulcer Spontaneous Cure Case Report*Read before the Chattahoochee Valley Medical Society, Alexander City, La., July 13, 1916.

**Published:** 1916-10

**Authors:** W. C. Gewin

**Affiliations:** Birmingham, Ala.


### ULCER: SPONTANEOUS CURE CASE REPORT.*

By W. C. Gewin, M. D., Birmingham, Ala.

Gastro-colic fistula is a comparatively rare condition. Helgenreimer (1) states in 1912 that the number of cases reported up to that time was 119.

Considering the relative frequency of chronic ulcers on the greater curvature and posterior surface of the stomach, and the close proximity of the transverse and first part of the descending colon to the stomach, the explanation of how a communication can occur between the stomach and the colon through a gastric ulcer is very apparent.

History of Case: C; male; age 36; white, farmer by occupation. Until about two years ago was a strong well nourished man weighing about 170 pounds. About two years ago he began having trouble with his digestion. The first symptom was eructation from the stomach beginning immediately after the ingestion of food. This was followed in a few days by a severe burning in the epigastrium, sour stomach, pain radiating to left of spine, etc. Because of the extreme discomfort excited by the taking of food, he was forced to live on not only a restricted but very sparing diet, consequently he became very thin and emaciated.

---

*Read before the Chattahoochee Valley Medical Society, Alexander City, La., July 13, 1916.

The symptoms and condition remained more or less constant until a few days before he came to the hospital for treatment, at which time he had a very profuse hemorrhage from the stomach. He said that the hemorrhage was so severe that he almost fainted and was so weak that he could not walk.

Examination: A thin, lanky, emaciated individual giving a very definite history of gastric ulcer. Marked tenderness over the stomach area. Because of his weakened condition, and the fact that he had had such a profuse hemorrhage within the last few days it was thought unwise to fill his stomach with a bismuth meal or to introduce a stomach pump.

He was given a concentrated liquid diet, and small nutritive enemas in an attempt to prepare him for the ordeal of a routine stomach examination.

His improvement was more rapid than would be expected considering his condition and past history. After he was at the Hospital about one week it was decided to give him a bismuth meal, and make observations under the fluoroscope, which was done by our radiologist, Dr. Weed. It was noticed that the opaque mixture on entering the stomach apparently passed right on through the posterior wall into the peritoneal cavity. Plates were made immediately afterwards which showed that the barium was actually passing through a fistulous opening. Because of the exhaustion incident to the examination it was not thought prudent to make any further examination at that time.

The only logical conclusion that we could come to was that the fistulous tract was between the stomach and colon.

Realizing the probability of exciting diarrhoea, fecal vomiting, etc., should strong food be thrown directly into the colon from the stomach, a liquid and soft diet was given him together with small nutritive enemas. He was also given large doses of sodium bicarbonate to counteract the acidity of the stomach.

His improvement was almost phenomenal and in the course of a couple of weeks his diet was graduallv increased. The increase of diet caused him no trouble, and he increased in weight rapidly.

It would have been very interesting to make another X-Ray examination after he was put on full diet to see what had become of the fistula, but unfortunately at that time we were installing new equipment and our X-Ray Department was temporarily out of commission.

As the man lives some distance from the city, we have been unable to make another X-Ray examination.

It is almost inconceivable that food could have passed directly from the stomach to the colon without causing irritation of the colon and diarrhoea. Also, how he could have gained in weight unless the food was passing through the small intestines. But as a matter of fact the X-Ray plates also show that food was passing through the pylorus into the duodenum. In my opinion the pyloric end of the stomach and the duodenum were in a perfectly healthy condition, and that the small intestine was performing its function normally, which explains why the man utilized his food properly. But why the ulcer should have healed and the fistula closed (which no doubt it did) is hard to explain in any other way except that we know that at least a large per cent. of gastro-enterostomy openings close after the gastric or duodenal ulcer has healed. Basing a deduction on the assumption that the theory of deficient local blood supply is partly responsible as an etiological factor in the formation of gastric ulcers, it is very possible that the healing of the ulcer was facilitated by the increased blood supply to the ulcer through the attachment of the affected area to the colon.

The only literature that I have been able to find on Gastrocolic fistulae is a short article by Hamann (2) which he read before the American Surgical Association, June 1915. He says: "The cause of these fistulae is nearly always some gastric affection, such as carcinoma or ulcer, which, secondarily, after adhesions to the colon have formed, results in perforation of the walls of the large intestine. Since the introduction of the operation of gastro-enterestomy, there has been added another factor, namely, gastro-jejunal or peptic ulcer of the jejunum, which may be described as an indirect fistula between the stomach and colon, the peptic ulcer of the jejunum perforating into the colon in close proximity to the gastro-enterostomy opening. However, the disturbance produced and the symptoms of such a gastro-jejuno-colic fistula are practically the same as those of a gastro-colic fistula."

"The chief symptoms of gastro-colic fistula are three in number, viz.: fecal vomiting or eructations of foul smelling (fecal odor) gases, without other signs of intestinal obstruction, diarrhoea, or lienteric discharges, and loss in weight. That these symptoms should occur is obvious and the explanation is equally plain. Other evidences of the condition are the ability to inflate the stomach from the rectum, the vomiting of enemas, the withdrawal by gastric lavage of colored fluid introduced into the rectum, and the finding of pepsin and hydrochloric acid in the feces. None of these symptoms are, however, constantly found."

In a recent communication the patient informs me that he has gained about forty pounds in weight, and that his indigestion is entirely relieved.

(1) Hilgenreiner: Central. f. die Grenzgeb. der Med. u. Chir., (2) Hamann: Annals of Surgery, August 1915, p. 208.

